# Texas

**Published:** 1896-06

**Authors:** 


*Texas.* The so-called Texas horsefly is reported to have already begun its disastrous season's work. A reliable remedy for the destruction of this parasite is much desired.

